# Malaria in children aged <6 months: a narrative review of current evidence, recommendations and practice gaps

**DOI:** 10.1186/s41182-026-00935-5

**Published:** 2026-04-24

**Authors:** Jéssica Dalsuco, Quique Bassat, Hazem Ahmed, Sabine Pfeffer, Cornelis Winnips, Caroline Boulton, Nekoye Otsyula, Umberto D’Alessandro

**Affiliations:** 1 Centro de Investigação Em Saúde de Manhiça (CISM), CISM, Rua 12, Vila da Manhiça, CP1929 Maputo, Mozambique; 2 ISGlobal, Hospital Clínic–Universitat de Barcelona, Barcelona, Spain; 3 ICREA, Passeig Lluís Companys 23, 08010 Barcelona, Spain; 4 Pediatric Infectious Diseases Unit, Pediatrics Department, Hospital Sant Joan de Déu (University of Barcelona), Barcelona, Spain; 5 CIBER de Epidemiología y Salud Pública, Instituto de Salud Carlos III, Madrid, Spain; 6 Novartis Pharma AG, Basel, Switzerland; 7 Novartis Pharma Services Inc., Nairobi, Kenya; 8 MRC Unit The Gambia at the London School of Hygiene and Tropical Medicine, London, UK

**Keywords:** Congenital malaria, Infants, Malaria, Neonatal malaria, Newborn

## Abstract

**Background:**

Although clinical malaria occurs far less frequently in newborns and young infants aged  <6 months than in older children, its importance and public health relevance should not be ignored. This population is often perceived to have protection against malaria, and making a diagnosis of malaria in this age group can be challenging, as the symptoms generally overlap with those of many other common conditions in infancy, possibly leading to misdiagnosis or treatment delay. This narrative review synthesises current evidence, explores existing treatment recommendations, and highlights practice gaps in malaria management in children aged <6 months (or weighing <5 kg).

**Methods:**

A literature search was conducted using PubMed, EMBASE, and Google Scholar and supplemented with World Health Organization and region-specific malaria treatment guidelines.

**Findings:**

Data on malaria prevalence in young infants are highly variable and heterogeneous, reflecting differences in the methodology used, diagnostic criteria and treatment approaches. Evidence regarding the safety and efficacy of available antimalarial therapies in this population is limited, and until recently, no antimalarial agents were licensed for neonates or infants weighing  <4.5 kg. Consequently, current recommendations may not adequately address the needs of this vulnerable group.

**Conclusion:**

Given the variability in the guidelines across different countries, there is an urgent need to make specific policy and practice amendments that can enhance understanding, bridge existing knowledge gaps and harmonise treatment approaches for at-risk populations worldwide.

**Supplementary Information:**

The online version contains supplementary material available at 10.1186/s41182-026-00935-5.

## Background

### Epidemiology

Malaria continues to have a devastating effect on global public health, posing a risk to the survival of people living in endemic regions. Although progress is being made towards reducing the disease burden, malaria continues to have a substantial impact on the health of individuals. According to the World Health Organization (WHO), an estimated 282 million cases of malaria were reported globally in 2024, resulting in 610,000 associated deaths [1].

Malaria is a vector-borne disease caused by one of the five *Plasmodium* species infecting humans, among which *Plasmodium falciparum* and *Plasmodium vivax* are responsible for most of the malaria-related deaths [2]. Young children and pregnant women are particularly vulnerable to malaria infection and mortality [3].

In 2020, an estimated 67.7 million cases among 247.7 million pregnancies were at risk of malaria transmission caused by *P. falciparum* and *P. vivax* [4]. In 2024, the WHO African Region continued to bear the greatest burden of malaria globally, accounting for 94% of cases and 95% of malaria-related deaths worldwide, with more than 75% of these deaths occurring in children aged  <5 years [1]. Within this region, approximately 36 million pregnancies were reported across 33 countries with moderate-to-high malaria transmission in this region, of which 13 million pregnancies (36%) were affected by malaria infection [1].

### Immunity and outcomes of malaria in pregnancy

In malaria-endemic areas with stable transmission, local populations gradually develop partial immunity to malaria through continuous exposure to the parasite over time [5]. Infants aged <6 months are generally considered to have protection or to be at a low risk of developing clinical malaria due to maternal antibodies and other intrinsic protective factors such as foetal haemoglobin (HbF) [6, 7]. However, when the maternal antibodies and HbF wane around 6 months after birth, young children become increasingly vulnerable to malaria infection and gradually acquire clinical immunity through repeated exposure to the parasite [6, 7]. Retrospective analyses have shown that the intensity of malaria transmission and, consequently, the level of exposure to the parasite may influence the level and function of malarial antibodies in young infants [8, 9].

Malaria during pregnancy can lead to adverse outcomes such as low birth weight (<2.5 kg) due to premature delivery or intrauterine growth retardation, both of which can lead to a marked increase in the risk of infant mortality. Moreover, it may also result in congenital malaria (CM) [10]. Primigravidae, secundigravidae and women living with human immunodeficiency virus (HIV) infection are at the highest risk of such adverse outcomes [10–13].

### Preventive measures

Despite the implementation of effective preventive measures targeting both pregnant women and children—such as insecticide-treated nets (ITNs), intermittent preventive treatment in pregnancy (IPTp) and seasonal or perennial malaria chemoprevention (PMC)—various challenges continue to exist in the diagnosis and treatment of malaria in children. Therefore, newer treatment interventions are needed for clinical malaria in very young children whenever these episodes occur.

In this regard, a better characterisation of malaria in infants, particularly among the youngest age groups, appears to be an important step for improving disease surveillance and developing control strategies tailored to this age group.

This narrative review describes the disease characteristics in infants aged <6 months, provides updates on the disease epidemiology and risk factors, and discusses the current clinical challenges and preventive measures, thereby highlighting the pressing need for evidence-based treatment recommendations for this age group.

## Methods

### Literature search strategy and selection criteria

To collect relevant articles, a literature search was performed on PubMed (until August 2025), EMBASE and Google Scholar using broad key words along with the Boolean operator 'AND' and 'OR'. The key words used for the search were 'congenital malaria', 'neonatal malaria', 'young children', 'infants', 'parasite', 'severe malaria', 'uncomplicated malaria', 'burden malaria', 'anaemia malaria', 'pregnancy', 'treatment', 'antimalarials', 'diagnosis', 'artesunate', 'artemether', 'artemether–lumefantrine', 'artesunate–mefloquine', 'artesunate–sulfadoxine–pyrimethamine', 'artesunate–sulfadoxine–amodiaquine', 'dihydroartemisinin–piperaquine', 'chloroquine', 'intermittent preventive treatment', 'perennial malaria chemoprevention', 'prevention', 'malaria vaccine', 'epidemics', 'mortality', 'pharmacokinetics' and 'pregnancy'.

The reference lists of the retrieved papers were screened for additional studies, with priority given to PubMed reviews. Other sources included region-specific malaria treatment guidelines, international guidelines from the WHO and other scientific websites covering the topic. The searches were conducted in English; only studies involving human subjects were selected.

### Extraction and analysis of the literature

Potentially relevant articles were identified, screened and synthesised by three authors (HA, NO and JD), and the following subtopics were identified: immunity, epidemiology, clinical presentation, risk factors, treatment, prevention and major knowledge gaps. Expert consensus was sought from JD, QB and UD.

## Characterisation of malaria in infants aged <6 months

The neonatal period refers to the first 6 months of life that extend into early infancy [14, 15]. Considering standard weight according to age growth charts, neonates and infants generally weigh <10 kg [16]. Malaria in infants is classified according to the time of infection. It can either be CM or neonatal malaria (NM) [Box 1]. Differentiating between CM and acquired NM can be challenging, especially in areas of intense malaria transmission [17].

**Box 1:** Glossary of terms

**Congenital malaria**	Presence of asexual *Plasmodium falciparum* parasites in cord blood or peripheral blood during the first week of life, following vertical transmission from the mother to the offspring. These infections are considered vertically transmitted because the time between the inoculation of the sporozoite and the appearance of blood-stage parasites exceeds 1 week [18, 19]
**Neonatal malaria**	Malaria infections occurring within 8–28 days of life typically caused by an infective mosquito bite after birth [19–21]

## Role of immunity

The risk of malaria in infants aged <6 months is relatively low owing to several innate and acquired factors from the mother [22]. Protective factors include maternal immunoglobulin G (IgG) antibodies, lactoferrin and immunoglobulin A (IgA) secreted in breastmilk. Breastfed infants benefit from a lower level of para-aminobenzoic acid (PABA) and a higher concentration of HbF, which inhibit malarial parasite growth in red blood cells [6, 7].

Breastmilk and transplacental route are two potential routes that have been postulated for the transfer of maternal antibodies to the infant. A study of 144 mother–child pairs including babies aged 2–12 months showed that IgA antibodies in the breastmilk play an important protective role for young infants; specific antibodies have been reported to act against the parasite ring stage, trophozoites, schizonts and gametocytes. However, the mechanism underlying such protective effects remains unclear, as IgA exerts mucosal immunity rather than humoral immunity [23]. Lactoferrin, a breastmilk component, seems to exert similar inhibitory effects on malarial parasites [23].

Studies of maternal and cord blood have suggested that placental transfer of antibodies may play a vital role in malaria prevention in young infants [24]. The generated antibodies play a protective role in the first 6 months of life, especially against the merozoite surface protein-1 (MSP1) glycosylphosphatidylinositol-anchored 19-kDa fragment (MSP1_19_) and the cysteine-rich interdomain region of the *P. falciparum* 732*var* gene (CIDR1α), as well as for anti–*P. falciparum*–specific IgG2 [6, 7]. The intensity of malaria transmission may play a role in the duration and efficiency of the protective effect by the maternal antibodies. In Western Kenya, infants from high malaria transmission areas had a higher initial MSP1 (a 42-kDa fragment)-specific antibody level and a longer period of the protective effect than those from low malaria transmission areas. In this time-to-infection analysis, no difference was observed between the low and high transmission areas. Despite this, a higher number of children in the low transmission areas were infected earlier. These results are reflective of the complex interplay of multiple factors including exposure, passive immunity from maternal antibodies and waning antibody levels with age [9].

HbF may contribute to innate immunity in young patients but does not directly inhibit parasite growth. Instead, it may act synergistically with IgG antibodies to reduce the ability of the parasitised red blood cells to cytoadhere to the microvasculature [25, 26]. Cytoadherence is an important process in the pathogenesis of severe malaria, and HbF and IgG dynamics may explain why infants and neonates are relatively well protected against severe malaria [25].

Finally, *Plasmodium* parasites, unlike humans, can synthesise essential folate from PABA through de novo synthesis, unlike humans. Breastfed infants typically have low PABA levels, which may contribute to the clearance of malarial parasites. This hypothesis is supported by findings from an in vivo study using mice infected with *Plasmodium yoelii*, a malarial species infecting rodents. Mice maintained on a PABA-deficient diet survived otherwise lethal infections, while those on normal diets did not, highlighting the role of dietary PABA in parasite survival [27].

The level of protection conferred through these pathways in young infants could result in asymptomatic and transient parasitaemia. Indeed, the rate of clinical malaria cases is lower in young infants (aged <6 months) than in older infants. In a study conducted in Nigeria, clinical malaria accounted for 4.4% of all illnesses that occurred in the first month of life; this figure increased steadily to account for 42.4% of all illnesses that occurred in infants aged 6 months [28].

## Prevalence of malaria in infants aged <6 months

The prevalence of malaria in infants aged < 6 months is poorly reported. Data for this demographic are not specified in routine programme reporting. Available data come from studies that have used varied methodology and objectives, enrolling different subgroups within this population with or without clinical symptoms. Moreover, variations in maternal immunity according to local endemicity, differences in diagnostic methods and environmental differences have led to varying outcomes even within the same country [29]. Such variation yields data that are both highly variable and heterogeneous [21, 30–33].

Two recent meta-analyses assessing the malaria prevalence during the first month of life corroborated the expected observation that prevalence patterns are influenced by both transmission intensity and the endemicity of the setting. The first meta-analysis, comprising 24 studies focused exclusively on CM—defined as the presence of parasites in peripheral or cord blood within the first 7 days of life irrespective of symptoms—assessed data from 8148 newborns and reported a global prevalence of 6.9% (95% CI 4.8–7.9%). Marked inter-country variability was observed, with estimates ranging from 0.0% in Colombia to as high as 46.7% in Nigeria [31]. As anticipated, the incidence of CM was significantly higher in areas of unstable malaria transmission than in those of stable malaria transmission (*P* = 0.035) [31], underscoring the role of partial protective immunity in reducing the risk of developing clinical CM [5, 34].

The second systematic review and meta-analysis examined the prevalence of symptomatic CM and NM in symptomatic young infants, thereby providing a clearer picture of the true clinical impact and resource needs for malaria in this age group [21]. It included 22 studies—primarily prospective—encompassing 28,083 neonates across 14 malaria-endemic countries. In this review, NM was defined as the presence of positive parasitaemia with asexual forms of the parasite associated with at least one symptom (fever, jaundice, anaemia, splenomegaly, vomiting, hepatomegaly, diarrhoea, restlessness, drowsiness, convulsions, poor feeding, cyanosis, pallor and respiratory distress) in infants aged <28 days. CM was defined as the presence of malaria in newborns aged up to 7 days. As expected, the overall crude prevalence of CM was 4% (95% CI 1.96–6.77) and lower than that reported in the previous review, and that of NM was 1.2% (95% CI 0.14–3.03). Due to limited data, no apparent differences were observed between point estimates from pooled studies from sub-Saharan Africa and those from other continents [21].

It is worth noting that the key limitation in the two meta-analyses is the difficulty in correlating the malaria infection with the clinical disease in this specific age group. In malaria-endemic regions, making a diagnosis of malaria in an ill newborn requires a comprehensive exclusion of other potential causes. Moreover, these studies have predominantly focused on a single continent (Africa) and species (*P. falciparum*), and very few studies (and data) focusing on other species or other malaria-endemic regions have been included in these reviews. Nonetheless, such studies may help call attention to the threat of CM and NM, which is often overlooked due to their perceived infrequency [34].

Even within countries, the estimated prevalence of CM can vary significantly. A recent systematic review evaluating 12 studies conducted across Nigeria—a country with a considerable malaria burden—reported prevalence estimates ranging from 5.1% to 96.3%, largely attributed to differences in transmission intensity and diagnostic approaches [35]. However, the review did not detail the specific diagnostic methods employed, limiting the ability to determine how many cases would have been detected through routine procedures such as microscopy or rapid diagnostic tests. Furthermore, although the review included studies published from the inception of each database through June 2024, it did not assess temporal trends in CM prevalence.

While Africa accounts for most global malaria cases, regions across Asia and South America continue to experience substantial disease impact. A meta-analysis of 14 studies (2009–2020) conducted in Colombia reported data from 1143 umbilical cord blood samples and 899 peripheral blood samples from symptomatic neonates [36]. The prevalence of CM was 1.3% (95% CI 0.6–3.0) as assessed by microscopy (thick blood smear [TBS]) and 16.2% (95% CI 8.2–29.5) as assessed by polymerase chain reaction (PCR) assay. The prevalence of submicroscopic CM (i.e. negative on TBS assessment and positive on PCR assay and based on three studies) was very high (22.0% [95% CI 13.2–34.3]). Most of the infections were due to *P. vivax*. The presence of submicroscopic parasitaemia prompts consideration of malaria as a potential contributor to the episode of ill health and indicates that re-testing neonates with undetermined causes of illness may be beneficial.

The studies included in these reviews are included in Additional File 1.

Few studies have focused on the entire <6-month-old age group, rather than CM or NM, likely due to the belief that these young infants have immunity to malaria leading to a lower research prioritisation. A cross-sectional study, conducted at the peak of the malaria transmission season in three countries in West Africa [30], evaluated the overall prevalence of malaria among infants (≤6 months old; *n* = 2219) across different transmission settings, comparing this to the prevalence in controls aged 1–9 and 10–15 years. The prevalence ranged from 3.7% to 21.7% by PCR assay. As expected, infants aged <6 months were less likely to have malaria than older children. However, within this subgroup aged <6 months, those weighing <5 kg had a significantly higher likelihood of malaria infection than those weighing >5 kg (adjusted odds ratio, 3.45, 95% CI 2.22–5.26; *P* = 0.001). This higher likelihood was potentially driven by limited coverage with available preventive measures [30].

It is important to note that the risk of malaria in young infants aged <6 months is not uniform and varies with age. This finding is demonstrated in a study conducted in Manhiça, Mozambique, between June 2003 and May 2005, wherein 30.5% (28,963 of 94,940) of the attendances to the outpatient clinic came from children due to clinical malaria. Infants aged <1 month represented only 0.14% (42 of 28,877) of these visits, while 1- to <6-month-old infants represented 3.52% (1019 of 28,877) of the visits [37]. During the same study period in Manhiça, among 8311 hospitalised children aged <15 years, 4080 (49.1%) had clinical malaria and 1100 (27%) had severe malaria. Severe malaria occurred very rarely in the neonatal period, with only a few cases having been diagnosed, but it was relatively frequent in the first 6 months of life, accounting for nearly 10% of all cases [38].

The studies discussed here have used diverse methodologies, varying diagnostic criteria and diverse treatment approaches, making direct comparison of the results challenging. However, they all point to a disease burden that should be addressed.

## Clinical features and outcomes

Several clinical symptoms are observed in children aged <6 months with malaria, including fever (the most common one), anaemia, jaundice, splenomegaly or heart galloping, prostration, refusal to eat, difficulty breathing, irritability, abdominal distension, diarrhoea, severe dehydration and coma [39–43]. However, the clinical presentation is often non-specific and may overlap with that of other infectious diseases such as sepsis and pneumonia, thus making its differential diagnosis highly challenging [39, 41, 43–45].

**Fig. 1:**
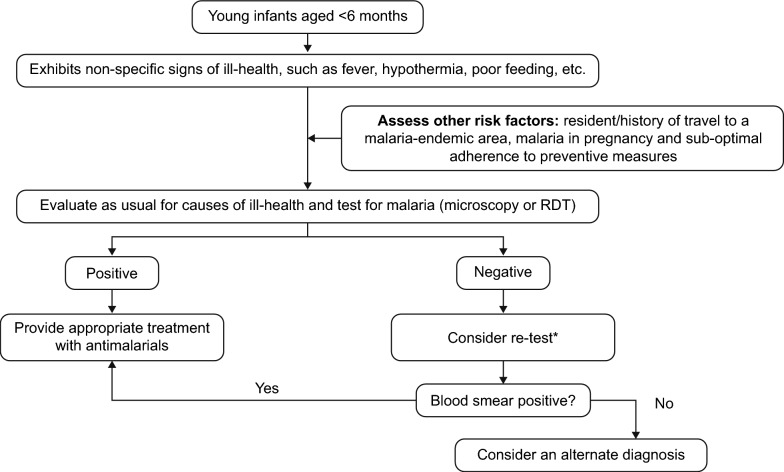
Algorithm for diagnosing malaria in young infants. This algorithm illustrates the recommended steps for diagnosing malaria in infants younger than 6 months. ^*^For suspected severe malaria, the WHO recommends testing blood films every 6–12 h or using a RDT [3]. Because young infants can present with lower parasitaemia, clinicians should consider re-testing when a negative test result is obtained but malaria is suspected in a young infant. In addition, where microscopy is the diagnostic standard, treatment should be provided to patients with a positive test result, irrespective of parasite density. *RDT,* rapid diagnostic test; *WHO,* World Health Organization

Considering the non-specific presentation of malaria in infants, clinical differentiation between malaria and sepsis in the absence of testing is difficult [40, 43–45]. Reports of a few infants presenting with malaria and sepsis concurrently are available, but some infants had an initial diagnosis of sepsis and a final diagnosis of malaria [40, 44, 45]. In areas of low endemicity, there is a higher likelihood that the malaria infection becomes symptomatic and progresses towards severe malaria due to treatment delays because of the low index of suspicion among clinicians [29, 44]. Figure 1 shows a simplified algorithm for diagnosing malaria in infants aged <6 months.

For over a decade, the WHO has recommended prompt parasitological confirmation of suspected malaria cases in infants before treatment [3]. Accurate diagnosis is particularly important in the most vulnerable population groups, such as young children, in malaria-endemic areas [3]. The current gold standard approach for malaria diagnosis is microscopy of Giemsa-stained blood slides [46, 47], although rapid diagnostic tests are also recommended [3]. For clinical cases typically associated with high-density parasitaemia, parasitological confirmation provides a straightforward diagnosis.

A birth cohort study in Kintampo, Ghana (*N* = 1819), suggested that infants in malaria-endemic areas have diverse infection patterns throughout their first year of life. As anticipated, while the first microscopic infections were detected as early as 1 month of age, the first symptomatic malaria episodes emerged from the age of 2 months onwards, with the median age at onset being 6 months (interquartile range: 5–8 months). Parasite densities were significantly higher in symptomatic infections than in asymptomatic ones, and infants aged > 6 months had greater densities than younger infants (*P* < 0.001) [48]. These observations are consistent with those of previous reports in the literature [30, 49].

The clinical presentation, diagnosis and treatment of selected cases in children aged <6 months or weighing <5 kg are summarised in Additional File 2.

## Risk factors and their associations

Multiple complex factors, including maternal immunity against malaria, contribute to the development of symptomatic CM [29, 50].

Regardless of gravidity, clinical malaria during pregnancy, placental malaria infection, HIV infection, travel by non-immune pregnant women to endemic areas and maternal anaemia are some of the predisposing/risk factors for CM [29, 50].

A systematic review and meta-analysis of studies with at least 12 months of postnatal follow-up revealed that malaria during pregnancy is significantly associated with an increased risk of *P. falciparum* parasitaemia (adjusted hazard ratio, 1.46; 95% CI 1.07–2.00; *P* < 0.001) and clinically defined malaria (adjusted odds ratio, 2.82; 95% CI 1.82–4.38; *P* < 0.001) in young children [51].

First pregnancy is the strongest risk factor for malaria in pregnant women, with the risk decreasing in successive pregnancies due to acquired immunity that prevents parasite adherence to the placenta [24, 52]. Ultimately, the immunological status of the newborn and disease outcomes are dependent on the complex interplay between maternal immune modulators, foetal immune immaturity and placental integrity [11, 24, 52].

Maternal anaemia can heighten the probability of bidirectional materno-foetal exchange by exerting stress on the placenta, which frequently results in increased placental weight through enhanced cellular proliferation, placental architectural remodelling, and angiogenesis [53].

Mothers infected with HIV experience a modification/decrease in the transfer of antibodies against some antimalarial antigens in the umbilical cord blood, predisposing the infant to a greater susceptibility to malaria [13], especially if they had an extremely low CD4 count (< 200 cells) with a relatively high parasite density [54].

Pregnant women living in non-endemic regions generally lack acquired immunity to malaria; therefore, travel to malaria-endemic areas is associated with an increased risk of severe disease and adverse maternal outcomes, including low birth weight and foetal loss [50].

**Fig. 2:**
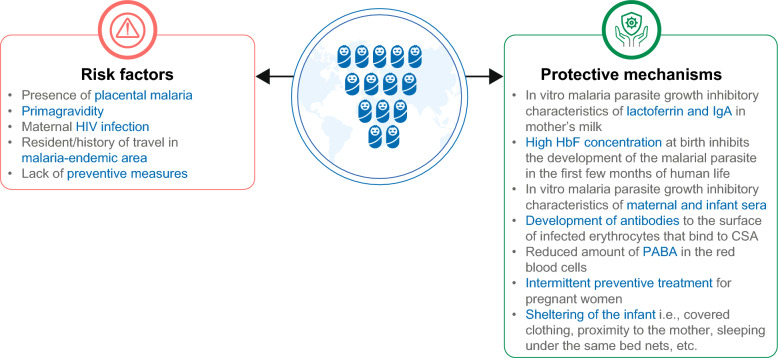
Risk and protective factors for malaria in neonates or infants. The figure depicts the factors that can pose risks for neonates or infants towards developing clinical malaria. On the contrary, protective mechanisms also confer protection against malaria in this vulnerable population. *CSA,* chondroitin sulphate A; *HbF,* foetal haemoglobin; *HIV,* human immunodeficiency virus; *IgA,* immunoglobulin A; *PABA,* para-amino benzoic acid

Figure 2 provides an overview of the key risk factors [29, 53–55] and protective mechanisms in neonates and young infants [6, 7, 18, 25, 34].

## Treatment options

As symptomatic malaria is uncommon in neonates, research on its optimal management remains limited, and treatment protocols are not well defined. This gap has resulted in a lack of evidence-based treatment recommendations for young infants [22].

Table 1 presents a summary of the malaria treatment guidelines for infants aged <6 months or weighing <5 kg recommended by the WHO and those adopted by various African countries; however, these national guidelines have considerable variability [3, 56–66].

**Table 1:** Recommended treatment guidance for the management of uncomplicated malaria in infants

Organisation/country	Treatment recommendations	Minimum age/BW covered	Comments/interpretation
WHO [3]	• Infants weighing <5 kg with uncomplicated *P. falciparum* malaria should be treated with an ACT at the same mg/kg BW target dose as that for children weighing 5 kg^a^ • Paediatric formulations and strengths should be used whenever possible	<5 kg	Not evaluated using the GRADE framework, as the available data were not suitable for assessment with the GRADE methodology
Burkina Faso [56]	• Oral AS-AQ or AL (fixed combinations): preferred treatment option • WBD is recommended for infants weighing ≥4.5 kg	≥4.5 kg	No guideline recommendations for young infants weighing <4.5 kg
Cameroon [57]	• Breakable/dispersible paediatric formulations • WBD is recommended for infants weighing ≥4.5 kg (AS-AQ) and ≥5 kg (AL)	2 months/ ≥4.5 kg	No guideline recommendations for young infants weighing <4.5 kg
Democratic Republic of the Congo [58]	• Use chloroquine/quinine or clindamycin for >1 month or weighing <5 kg BW (if no other alternatives are available)	2 months/ <5 kg	Utilises non-ACTs as on-label treatment for young infants
Ghana [59]	• AS-AQ or AL: first-line treatment • DHAP: second-line option • Treat infants weighing <5 kg with uncomplicated *P. falciparum* malaria with ACT at the same mg/kg BW target dose as that for children weighing 5 kg	<5 kg	Aligned to the WHO recommendation
Mali [60]	• AL: preferred first-line treatment • Dispersible tablets to be used for children aged 2 months to 6 years (5–24 kg BW)	2 months/5 kg	No guideline recommendation for young infants weighing <5 kg
Mozambique [61]	• IV artesunate with oral follow-on therapy • AL: first-line treatment • AS-AQ: alternative first-line option	<5 kg	Malaria in this age group is automatically considered to be severe
Niger [62]	• AL: first-line treatment • DHAP: alternative first-line option	Not specified	No guideline recommendation for young infants weighing <5 kg
Nigeria [63]	• AL, AS-AQ, DHAP and PA are the recommended first-line treatments	Not specified	No guideline recommendation for young infants weighing <5 kg
Uganda [64]	• AL: first-line treatment • AS-AQ: alternative first-line option • DHAP: second-line option (use quinine tablets if unavailable)	<4 months	Off-label
United Republic of Tanzania [65]	• AL: preferred treatment option • DHAP/AS-AQ: recommended as alternatives, where there is no response to AL, or it is contraindicated • WBD is recommended in patients weighing >5 kg	For AL and DHAP: 5 kg; For AS-AQ: 2 months/ ≥4.5 kg	Aligned to the WHO recommendations
Sudan [66]	• AL: first-line therapy in the tablet form • DHAP: second-line therapy in the tablet form • Dispersible tablets are available for the paediatric population • WBD recommendations are provided for those weighing ≥5 kg	<5 kg (dosing needs to be adjusted per the BW)	Aligned to the WHO recommendations

^a^Treatment response should be monitored closely

*ACT,* artemisinin-based combination therapy; *AL,* artemether–lumefantrine; *AS-AQ,* artesunate–amodiaquine; *BW,* body weight; *DHAP,* dihydroartemisinin/piperaquine; *GRADE,* Grading of Recommendations Assessment, Development and Evaluation; *PA,* pyronaridine–artesunate; *SPAG,* sulfadoxine pyrimethamine–amodiaquine; *SPAQ,* sulfadoxine pyrimethamine–amodiaquine; *WBD,* weight-based dosing; *WHO,* World Health Organization

For severe malaria, where parenteral therapy cannot be immediately administered, pre-referral treatment with rectal artesunate (a single dose of 10 mg/kg body weight [BW]) is recommended in children aged <6 years [3, 67], on the assumption that these children would be referred and started with parenteral artesunate. For the latter, to ensure equivalent exposure to the drug, the WHO recommends treating children weighing <20 kg with a higher mg/kg BW dose of artesunate (3 mg/kg) than those weighing  >20 kg and adults (2.4 mg/kg) [3].

Only a few studies have focused on infants weighing <5 kg, and currently available artemisinin-based combination treatments (ACTs) are licensed for use only in patients weighing  >4.5 kg or >5 kg [22]. A recently concluded phase 2/3 study recruited infants weighing <5 kg with malaria parasitaemia and evaluated a physiologically based pharmacokinetic (PK) dose-optimised artemether/lumefantrine (5/60 mg) [68]. This study was triggered by a PK study evaluating standard doses of artemether/lumefantrine, which reported a 2- to 3-fold elevation in artemether C_max_ in infants weighing <5 kg, when compared with historical references in children weighing >5 kg [69]. Preclinical studies have shown neurotoxic effects of high artemether exposures [70–72].

In addition, many trials of antimalarial drugs have excluded infants aged <6 months, reporting little or no information to guide dosing recommendations for this age group [18, 22]. Infants differ physiologically from older children and adults in terms of body composition, organ maturation and metabolic processes, which influences the PK and pharmacodynamics of antimalarial drugs [73]. Thus, dosing strategies based on BW and allometric scaling—commonly used in older children—may not be appropriate for this age group [22]. For instance, a previous study demonstrated that standard parenteral quinine dosing in children aged <2 years with severe malaria resulted in elevated drug levels, with an increased risk of cardiotoxicity [74].

While failure to treat malaria in young infants can have fatal consequences, it can also lead to other consequences such as severe anaemia, seizures and coma [3, 41]. However, it is also crucial to understand that organs continue to mature in the early postpartum period, and this has an impact on the distribution, disposition and, therefore, safety and effectiveness of the drugs administered to young infants [75], warranting dedicated studies for identifying appropriate dosing regimens for the optimal exposure of antimalarials in this age group.

## Existing preventive measures

The prevention of malaria during pregnancy remains the cornerstone strategy for reducing the incidence of CM in infants. Pregnant women must be routinely screened for malaria infection, and if the test results are positive, they should be treated promptly and effectively.

Prevention of malaria in non-immune pregnant women travelling to endemic areas is challenging. Therefore, it is recommended that pregnant women should avoid non-essential travel to areas where malaria is prevalent [50].

The WHO recommends that pregnant women living in malaria-endemic areas should be protected against infection by using ITNs and receiving IPTp with sulfadoxine–pyrimethamine [3].

Table 2 outlines the preventive interventions that are currently recommended by the WHO [3, 76].

**Table 2:** Malaria prevention strategies for young infants at risk

Preventive measure	WHO recommendation [3]
1. ITNs/LLINs^a,b^	• Use pyrethroid–chlorfenapyr ITNs instead of pyrethroid-only LLINs for malaria prevention where pyrethroid resistance exists *(Strong recommendation, 2023)* • Pyrethroid-only LLINs should be deployed for the prevention and control of malaria living in areas with ongoing malaria transmission *(Strong recommendation, 2019)*
2. IRS	• Use IRS for the prevention and control of malaria in children living in areas with ongoing transmission *(Strong recommendation, 2025)*
3. IPTp	• IPTp-SP should be given to all pregnant women residing in • malaria-endemic regions, regardless of gravidity, beginning in the second trimester^c^ *(Strong recommendation, 2022)* • Administer at least three doses during pregnancy at predetermined intervals to reduce the disease burden and adverse pregnancy/birth outcomes
4. PMC (formerly IPTi)	• ITP-SP is recommended for children up to 2 years old who are at a high risk of severe malaria *(Conditional recommendation, 2022)* • Target age groups for chemoprevention should be tailored based on local data; while IPTi was originally targeted at infants aged < 12 months in response to a high disease burden in this group, research shows benefits for children aged up to 24 months
5. SMC	• Advised in regions with seasonal malaria transmission, involving intermittent antimalarial treatment for children at a high risk of malaria during peak transmission *(Strong recommendation, 2022)* • SP-AQ is currently recommended, given every 28 days starting at the beginning of the transmission season for three to five cycles, depending on local conditions [77] • Dosing schedules [77]: *Infants aged 3 to* < *12 months:* ▪ Day 1: one tablet SP (250/12.5 mg) + one tablet AQ (75 mg) ▪ Days 2 and 3: one tablet AQ each day *Children aged 12–59 months:* ▪ Day 1: one tablet SP (500/25 mg) + one tablet AQ (150 mg) ▪ Days 2 and 3: one tablet AQ each day
6. PDMC	• Children admitted to the hospital with severe anaemia in malaria-prone areas should receive a full course of antimalarial medicine at set times after hospital discharge to lower the risk of re-admission and death *(Conditional recommendation, 2022)* • Alternative medicines to first-line malaria treatment are preferred. Dose by weight when possible; use age-based dosing only if the weight is unknown
7. Vaccines	• The two malaria vaccines (WHO-prequalified), namely RTS, S/AS01 (Mosquirix™) and R21/Matrix-M, are recommended starting from 5 months of age^d^^,e^ for the prevention of *P. falciparum* malaria in children living in malaria-endemic areas, prioritising areas of moderate and high transmission *(Strong recommendation, 2023)* • Administer as a four-dose schedule, ensuring a minimum interval of 4 weeks between each dose. For extended protection, the fourth dose should be administered 6–18 months after the third dose • A fifth dose may be administered 1 year after the fourth dose in areas with high seasonal malaria and considered elsewhere if the children remain at risk, depending on feasibility and cost-effectiveness • Can be administered to malnourished infants and children • RTS,S/AS01 may be administered for pre-term infants (born before 37 weeks of gestation), low BW and HIV-infected infants and children

^a^This is not an exhaustive list of WHO recommendations for this category

^b^ITNs prequalified by the WHO are preferred

^c^Not to be administered before week 13 of pregnancy

^d^Countries may administer the first vaccine dose before 5 months of age to improve coverage or effectiveness, based on operational needs

^e^Although the WHO prequalification issued for the RTS,S/AS01 and R21/Matrix-M malaria vaccines permits children to receive the first dose from 5 months of age, the RTS,S/AS01 manufacturer’s licensure specifies from 6 weeks to 17 months of age. Studies on RTS,S/AS01 indicated lower efficacy if the first dose was administered around 6 weeks of age. The efficacy of the RTS,S/AS01 and R21/Matrix-M vaccines is not expected to be significantly diminished when the initial dose is administered at 4 months of age rather than at 5 months

*AQ,* amodiaquine; *HIV,* human immunodeficiency virus; *IPTi,* intermittent preventive treatment of malaria in infants; *IPTp,* intermittent preventive treatment in pregnancy; *IRS,* indoor residual spraying; *ITN,* insecticide-treated net; *LLIN,* long-lasting insecticidal net; *PDMC,* post-discharge malaria chemoprevention; *PMC,* perennial malaria chemoprevention; *SP,* sulfadoxine–pyrimethamine; *SMC,* seasonal malaria chemoprevention; *WHO,* World Health Organization

## Discussion

This review summarises various aspects of malaria in children aged <6 months (or weighing <5 kg), including disease classification, updated data on prevalence from different regions, latest reports on infant immunity, risk factors, clinical challenges and existing preventive measures.

Due to the perceived rarity of malaria in newborns and the dearth of studies targeted to this population, data on malaria prevalence in neonates and young infants are limited [30]. The true impact of the disease may be underestimated or underreported in this age group due to the lack of routine screening for malaria in newborns with fever, low index of suspicion and absence of specific signs and symptoms, coupled with delayed symptom presentation [29, 30, 77]. To fully understand the disease burden and ensure proper diagnosis and treatment for young infants, we recommend systematic collection and evaluation of data on infection rates, clinical cases and risk factors, beginning with local epidemiological data.

Global malaria control efforts have achieved significant progress through WHO-recommended strategies such as vector control and preventive chemotherapies, which have substantially reduced the disease burden. A key measure recommended for malaria prevention in young infants and anyone living in a high transmission setting is sleeping under long-lasting insecticidal nets (LLINs) [78, 79]. In 2018, approximately 60% of pregnant women and children aged <5 years used LLINs/insecticide-treated bed nets [78]*.* UNICEF reports indicate that LLINs play a pivotal role in decreasing malaria incidence by 50% in sub-Saharan Africa [79].

Despite these achievements, gaps in coverage persist. Many individuals at risk still lack access to quality care and essential interventions. For instance, although IPTp is currently recommended in 34 African countries, only 45% of eligible pregnant women and girls received all three doses of IPTp in 2024 [1].

To enhance access to these interventions, it is essential to prioritise patient education, strengthen stock management, and implement innovative strategies for reaching patients in remote areas [1]. From a programmatic standpoint, this will necessitate targeted budgetary allocation.

Based on clinical trial evidence, malaria vaccines are recommended only for children aged >5 months [80–82]. In the phase 3 study of RTS,S/AS01 infants aged 6–12 weeks at the first vaccination had a weaker and less significant vaccine response in contrast to those aged 5–17 months at the time of the first vaccination [83]. In addition, current malaria vaccines are not recommended for use in pregnancy. Future vaccines should target these groups. Meanwhile, deployment of currently available public health interventions such as IPTp, PMC and use of LLITNs should be optimised to confer better protection to these groups.

While much is known about malaria management in infants and children with ≥5 kg BW, limited clinical data on malaria management are available for infants with <5 kg BW. The WHO recommends that infants diagnosed with malaria receive immediate treatment, as delays in managing *P. falciparum* can be fatal [3]. Current guidelines for malaria treatment in this population take the usual weight-based approach [3]. However, infants undergo significant physiological and anatomical changes during the first year of life, which may affect the exposure, safety and efficacy of treatments administered to them [22, 73, 75]. It is, therefore, necessary to ensure that individuals aged <6 months are appropriately represented in clinical trial populations and receive evidence-based safe and effective treatments.

## Conclusion

Malaria in infants under 6 months of age remains a significantly under-recognised and under-researched public health challenge. The true disease burden is likely underestimated owing to limited surveillance, non-specific symptomatology and a paucity of targeted research. While advances in malaria control—such as improved prevention tools, vaccines and expanded treatment access—have yielded encouraging progress, substantial gaps persist in the coverage and quality of interventions for this vulnerable age group. Current treatment recommendations remain pragmatic, relying on extrapolation from older children, highlighting the urgent need for evidence-based, age-specific guidelines.

Strengthening routine data collection, increasing representation in clinical trials, and improving coverage of proven preventive measures, including ITNs and preventive therapies for pregnant women, are critical steps forward. For clinicians in endemic regions and those managing at-risk migrant populations, heightened awareness and re-evaluation of current approaches may facilitate better diagnosis, management and, ultimately, reduction in malaria-related morbidity and mortality among young infants.

## Strengths

- The review identified and emphasised critical gaps in current research, especially those related to risk factors, transmission routes and clinical outcomes, for infants aged <6 months, who belong to a particularly vulnerable demographic.
- The inclusion of studies from diverse regions enriched the analysis by providing varied perspectives and contextual insights, which enhance the applicability and relevance of the findings.

## Limitations

- This study does not present a systematic review or a meta-analysis of all primary and secondary literature published on the relevant subject; therefore, the findings should be interpreted within this context.
- Outcomes associated with malaria in infants within this age group remain insufficiently documented, even in regions where malaria is endemic. This limitation restricts the ability to draw robust and definitive conclusions.
- By utilising and assessing technical reports, this review depends on original quality assessments conducted by the authors of these reports.
- The studies included in this review have employed a variety of methodologies, diagnostic criteria and treatment approaches. This diversity poses challenges for direct comparison and increases overall heterogeneity in the review.

## Supplementary Information

Additional file 1.

Additional file 2.

## Data Availability

No datasets were generated or analysed during the current study.

## Abbreviations

ACT: Artemisinin-based combination treatment
BW: Body weight
CD4: Cluster of differentiation 4
CIDR1*α*: Cysteine-rich interdomain region of the *P. falciparum* 732*var* gene
CM: Congenital malaria
HbF: Foetal haemoglobin
HIV: Human immunodeficiency virus
IgA: Immunoglobulin A
IgG: Immunoglobulin G
IPTp: Intermittent preventive treatment in pregnancy
ITN: Insecticide-treated nets
LLIN: Long-lasting insecticidal net
MSP1_19_: Merozoite surface protein-1 glycosylphosphatidylinositol-anchored 19-kDa fragment
NM: Neonatal malaria
PABA: Para-aminobenzoic acid
PCR: Polymerase chain reaction
PK: Pharmacokinetics
PMC: Perennial malaria chemoprevention
TBS: Thick blood smear
WHO: World Health Organization
