# Supplementary material for: Malaria in children aged <6 months: a narrative review of current evidence, recommendations and practice gaps
Source: Trop Med Health. 2026 Apr 24;54:94. doi: 10.1186/s41182-026-00935-5 (PMC13195890; doi:10.1186/s41182-026-00935-5)
Supplement: Supplementary file 1 — Additional file 1. [file 41182_2026_935_MOESM1_ESM.docx]

**Additional file 1**

**Table.** Prevalence of malaria in neonates or infants by country

| Country | Author, Year | Study period | Diagnostic method | Prevalence | Other relevant information |
| --- | --- | --- | --- | --- | --- |
| Columbia | Agudelo, 2013 [1] | 2008-2011 | Simultaneous application of microscopy and PCR | CM was absent | - |
|  | Agudelo-García, 2017 [2] | 2009-2014 | Microscopy and qPCR | Frequency of CPI: 27% (37/137) | - CPI was defined as the presence of *Plasmodium* in the umbilical cord blood not associated with clinical symptoms or signs^a^ - All cases of CPI were sub-microscopic, i.e., thick blood smear negativity and qPCR positivity |
|  | Carmona, 2009 [3] | 2005-2007 | Microscopy | CM: 2.7% (5/183) | - Among 220 pregnant women with GM, 183 infants were followed up during their first month of life |
|  | Carmona, 2010 [4] | 2004-2008 | Now ICT malaria Pf/Pv® and microscopy | CM: 1.82% (2/110) | - |
|  | Campos, 2011 [5] | NR | LM and nPCR | Frequency of cord blood parasitaemia: 2% (2/84) by LM assay and 13% (11/84) nPCR assay | - CM was defined as the presence of *Plasmodium* in the neonate’s umbilical cord blood, regardless of symptom presentation. For the purposes of this study, neonates were not followed up after birth - Sub-microscopic parasitaemia was identified in 11% of the umbilical cord samples |
|  | Piñeros-Jiménez, 2011 [6] | 2005-2007 | Microscopy | CM: 4.3% (5/116) | - CM was defined as either (1) a newborn from a MIP mother with the same *Plasmodium* species infection at birth or within 21 days, or (2) *Plasmodium* species infection of the umbilical cord |
|  | Arango, 2013 [7] | 2005-2011 | Microscopy and qPCR assay | Frequency of cord blood parasitaemia: 29% (28/96) | - |
| Burkina Faso | Cisse, 2016 [8] | 2010 | Microscopy | - Malaria parasite infection in newborns: 0.9% (3/320) - Congenital malaria parasitaemia among infants born to mothers with placental malaria parasitaemia: 10.3% (3/29) | - |
|  | Ouédraogo, 2012 [9] | NR | Microscopy | Umbilical cord parasitaemia: 1.4% (18/1309) in all newborns, 5.2% (11/211) in babies born from mothers with peripheral and placental malarial infection | - |
|  | Tiono, 2009 [10] | NR | Microscopy | Cord blood parasitaemia^b^: 1.41% (6/423) | - Pregnant women were randomised to receive one of the three treatment regimens for the prevention of placental malaria: classical chemoprophylaxis using CQ, IPT using CQ, and ITP using two doses of SP |
| Burundi | Stassijins, 2016 [11] | 2014 | RDT (SD-Bioline HRP2 and CareStart pan-pLDH), microscopy. qPCR assay was used for molecular diagnosis | CM was absent | - |
| Cameroon | Chiabi, 2012 [12] | 2007 | Microscopy | CM: 23.79% (54/227) | - |
| Côte d'Ivoire | Vanga-Bosson, 2011 [13] | 2008 | RDT was used for peripheral and placental blood. Positive RDTs were confirmed using LM | CM: 4.7% (4/85) (in infants born to mothers with placental malaria parasitaemia | - Neonatal blood samples were collected 2 hours after birth - CM was defined as the presence of asexual parasites in peripheral blood within the first day of life |
|  | Adja, 2009 [14] | 2005-2006 | Microscopy | NM: 0.98% (6/615) | - |
| Ghana | Enweronu-Laryea, 2013 [15] | 2008 and 2010 | Microscopy, RDT (The First Response^®^ malaria antigen pLDH/HRP2 Combo Test and nPCR assay was used for the amplification of the MSP gene | CM: 2.2% (9/405) | - |
| India | Singh, 2014 [16] | NR | Microscopy | CM: 2.95% (6/203)  NM: 8.4% (6/72) | - |
| Indonesia | Fitri, 2014 [17] | 2012-2013 | Microscopy; nPCR assay | CM: 42.4% (39/92) | - Samples were collected from a subgroup of newborns with an increased risk of CM at TC Hillers Hospital, Maumere |
|  | Jahja, 2014 [18] | 2012-2013 | Microscopy; nPCR assay | CM among all LBW newborns: 6.79% using microscopy and 7.78% using the nPCR assay | - Inclusion required pre-term delivery, LBW, neonatal illness within the first week (e.g. anaemia, poor feeding, jaundice, seizures, fever and hepatomegaly), and informed consent from the mother or guardian - CM was defined as an infection resulting from the transmission of live parasites from an infected pregnant woman to her foetus that occurs prenatally or during delivery and persists after birth. In addition, only asexual malarial parasites were present in the peripheral blood smear of the newborns during the first 7 days of life, irrespective of the clinical symptoms |
| Kenya | Mwaniki, 2010 [19] | 2002-2009 | Blood film parasitaemia | NM: 0.35% (18/5114)  CM: 0.21% (11/5114) | - CM was defined as symptoms attributable to only malaria with evidence of intra-erythrocytic asexual forms of *Plasmodium* species in the first 7 days of life - Neonatal malaria was defined as intra-erythrocytic asexual forms of *Plasmodium* species in the first 28 days of life in a sick^c^ neonate |
| Mali | Dicko-Traoré, 2011 [20] | 2006-2008 | RDT (Optimal IT^®^), LM and PCR | NM: Absent | - |
| Nigeria | Akindele,1993 [21] | 1993 | Microscopy | CM: 23.7% (14/59) | - |
|  | Ibhanesebhor, 1995 [22] | 1993 | Microscopy | NM: 8% (16/203) | - |
|  | Orogade, 2004 [23] | 2001-2002 | Microscopy | NM: 8.25% (17/206) | - |
|  | Obiajunwa, 2005 [24] | 1997 | Microscopy | CM: 46.7% (56/120) | - |
|  | Mukhtar, 2005 [25] | 2002 | Microscopy | CM: 15.3% (16/104) | - |
|  | Okafor, 2006 [26] | 2003-2004 | Microscopy | CMD: 3.7% (28/658) | - |
|  | Falade, 2007 [27] | 2003-2004 | Microscopy | CM: 5.1% (95/1875) | - |
|  | Sotimehin, 2008 [28] | 2004-2005 | Microscopy, RDT (OptiMAL^®^) | CM: 10.9% (21/192) | - |
|  | Orogade, 2008 [29] | 2003-2004 | LM | CM: 5.1% (95/1875) | - CM was referred to as the presence of malarial parasites in the newborn, regardless of symptoms |
|  | Ekanem, 2008 [30] | NR | Microscopy | CM: 13% (71/546) | - Neonates admitted to the neonatal unit who presented with clinical features suggestive of sepsis were included in the study |
|  | Lesi, 2010 [31] | 2002 | Microscopy | CM: 13.6% (14/96) | - |
|  | Oduwole, 2011 [32] | 2009 | nPCR | CM: 2.0% (4/204) | - CM was defined as malaria infection acquired by the transmission of malaria parasites from the mother to the child during pregnancy or perinatally during labour was determined with evidence of ring forms of malaria parasites in the cord blood smear at delivery or in the peripheral blood smear of an infant within the first 7 days of life, irrespective of clinical symptoms |
|  | Omalu, 2011 [33] | 2010-2011 | Microscopy | Cord blood parasitaemia: 5.92% (9/152) | - |
|  | George, 2013 [34] | 2010 | Microscopy | CM: 9.6% (27/281) | - |
| Peru | Silva, 2015 [35] | 2011-2013 | Microscopy | CM: 9.6% | - |
| Sudan | Omer, 2019 [36] | 2012-2015 | Microscopy and nPCR | Parasitaemia in cord blood samples: 8.1% | - The study evaluated only sub-microscopic malaria infections |
| Tanzania | Kitua, 1996 [37] | 1993-1994 | Microscopy | CM: 5.3% (3/56) | - CM was referred to as peripheral parasitaemia within 7 days of birth |
|  | Adachi, 2000 [38] | 1996 | Microscopy and PCR | CM: 0.33% (1/298) | - |
|  | Mosha, 2010 [39] | 2009 | Microscopy | CM: 4.0% (8/190) | - |
| The Gambia | Obu, 2011 [40] | 1998-1999 | Microscopy | CM: 35.7% (5/14) | - Neonates (age 0-28 days) admitted in the neonatal unit of the hospital with features suggestive of neonatal septicaemia were enrolled in the study |
| Togo | Balaka, 2000 [41] | NR | Microscopy | - CMD: 19% (40/2352) - CMI: 19.06% (114/598) | - CMD was diagnosed when clinical symptoms appeared alongside positive parasitaemia - CMI diagnosis was maintained when despite positive parasitaemia, no clinical manifestations were observed in the newborn |
| Zaire | Njirjesy, 1993 [42] | 1989-1990 | Microscopy | - Cord blood parasitaemia: 9% (26/297) - Neonatal blood parasitaemia: 7% (19/290) | - Neonatal samples were not obtained from the seven babies who survived for <48 hours |
| Multicentre study (Guatemala, Colombia, Brazil, India, and Papua New Guinea) | Bardají, 2017 [43] | 2008-2011 | Microscopy and RT-PCR | CM cases *(any Plasmodium species)*:   - In cord blood: 0.27% (11/4061) using microscopy and 5.2% (20/385) using PCR assay - In newborn blood: 0.07% using microscopy (3/4309) | - CM was defined as presence of asexual *Plasmodium* parasites of any species in the cord blood or in the newborn’s peripheral blood at delivery, regardless of clinical symptoms or signs in the neonate |

^a^If the definition is more stringent and requires simultaneous detection of parasites of the same species in both maternal and placental blood, the observed frequency of CPI in this study was 13%.

^b^Cord bloods were collected to assess for *P. falciparum* infection after childbirth.

^c^Hospitalised neonates were classified as sick.

CM, congenital malaria; CMD, congenital malaria diseases; CMI, congenital malaria infestations; CPI, Congenital plasmodial infection; CQ, chloroquine; GM, gestational malaria; HRP2, histidine rich protein; IPT, intermittent preventive treatment; LM, light microscopy; MIP, malaria in pregnancy; MSP, merozoite protein 2; NM, neonatal malaria; nPCR, nested polymerase chain reaction; NR, not reported; pLDH, parasite lactate dehydrogenase; qPCR, quantitative polymerase chain reaction; RDT, rapid diagnostic test; SP, sulphadoxine/pyrimethamine.

**References:**

1. Agudelo O, Arango E, Maestre A, Carmona-Fonseca J. Prevalence of gestational, placental and congenital malaria in north-west Colombia. Malar J. 2013;doi:10.1186/1475-2875-12-341.
2. Agudelo-García OM, Arango-Flórez EM, Carmona-Fonseca J. Submicroscopic and asymptomatic congenital infection by Plasmodium vivax or P. falciparum in Colombia: 37 cases with placental histopathology and cytokine profile in maternal and placental blood. J Trop Med. 2017;doi:10.1155/2017/3680758.
3. Carmona-Fonseca J, Amanda Maestre-B. Incidencia de las malarias gestacional, conge´ nita y placentaria en Uraba´ (Antioquia, Colombia), 2005–2007. Rev Colomb Obstet Ginecol. 2009;60(1):19-33.
4. Carmona-Fonseca J, Franco Gallego A, Arango-Flórez EM, Agudelo-García OM, Maestre Buitrago A. Now ICT malaria Pf/Pv® frente a microscopía (gota gruesa-extendido) para diagnóstico de malaria en Urabá (Colombia). Iatreia. 2010;23(2):137-45.
5. Campos IM, Uribe ML, Cuesta C, Franco-Gallego A, Carmona-Fonseca J, Maestre A. Diagnosis of gestational, congenital, and placental malaria in Colombia: comparison of the efficacy of microscopy, nested polymerase chain reaction, and histopathology. Am J Trop Med Hyg. 2011;84(6):929-35.
6. Piñeros-Jiménez JG, Álvarez G, Tobón A, Arboleda M, Carrero S, Blair S. Congenital malaria in Urabá, Colombia. Malar J. 2011;https://doi.org/10.1186/1475-2875-10-239
7. Arango EM, Samuel R, Agudelo OM, Carmona-Fonseca J, Maestre A, Yanow SK. Molecular detection of malaria at delivery reveals a high frequency of submicroscopic infections and associated placental damage in pregnant women from northwest Colombia. Am J Trop Med Hyg. 2013;89(1):178-83.
8. Cisse M, Diallo AH, Somé DA, Poda A, Awandare AG, Guiguemdé TR. Association of placental Plasmodium falciparum parasitaemia with maternal and newborn outcomes in the periurban area of Bobo-Dioulasso, Burkina Faso. Parasitol Open. 2016;doi:10.1017/pao.2016.12.
9. Ouédraogo A, Tiono AB, Diarra A, Bougouma EC, Nébié I, Konaté AT, et al. Transplacental transmission of Plasmodium falciparum in a highly malaria endemic area of Burkina Faso. J Trop Med. 2012;doi:10.1155/2012/109705.
10. Tiono AB, Ouedraogo A, Bougouma EC, Diarra A, Konaté AT, Nébié I, et al. Placental malaria and low birth weight in pregnant women living in a rural area of Burkina Faso following the use of three preventive treatment regimens. Malar J. 2009;doi:10.1186/1475-2875-8-224
11. Stassijns J, Van Den Boogaard W, Pannus P, Nkunzimana A, Rosanas-Urgell A. Prevalence and diagnostics of congenital malaria in rural Burundi, a crosssectional study. Malar J. 2016; doi:10.1186/s12936-016-1478-0.
12. Chiabi A, Lendem I, Kobela M, Mah E, Tietche F, Tchokoteu P-F. Incidence de paludisme congénital dans deux services de néonatalogie à Yaoundé, Cameroun. J Pédiatrie Puériculture. 2012;25(6):301-8.
13. Vanga-Bosson HA, Coffie PA, Kanhon S, Sloan C, Kouakou F, Eholie SP, et al. Coverage of intermittent prevention treatment with sulphadoxine-pyrimethamine among pregnant women and congenital malaria in Cte d’Ivoire. Malar J. 2011;doi:10.1186/1475-2875-10-105.
14. Adja E, Dick F, N'guessan R. Epidemiological study of the malaria at the neonatal period in the teaching hospital of Yopougon--Republic of Cote d'Ivoire. Le Mali Medical. 2009;24(3):36-9.
15. Enweronu-Laryea CC, Adjei GO, Mensah B, Duah N, Quashie NB. Prevalence of congenital malaria in high-risk Ghanaian newborns: a cross-sectional study. Malar J. 2013;12:17.
16. Singh J, Soni D, Mishra D, Singh HPBS. Placental and neonatal outcome in maternal malaria. Indian Pediatr. 2014;doi:10.1007/s13312-014-0402-3.
17. Fitri LE, Jahja NE, Huwae IR, Nara MB, Berens-Riha N. Congenital malaria in newborns selected for low birth-weight, anemia, and other possible symptoms in maumere, Indonesia. Korean J Parasitol. 2014;doi:10.3347/kjp.2014.52.6.639.
18. Jahja NE, Huwae IR, B Nara M, Harley A, Widaningrum T, Fitri LE. Comparison of nested polymerase chain reaction and microscopy as diagnostic tools in congenital malaria: a study at Tjark Corneile Hillers Hospital Maumere, Indonesia. Malays J Med Sci. 2014;21(5):17-23.
19. Mwaniki MK, Talbert AW, Mturi FN, Berkley JA, Kager P, Marsh K, et al. Congenital and neonatal malaria in a rural Kenyan district hospital: an eight-year analysis. Malar J. 2010;9:313.
20. Dicko-Traoré F, Sylla M, Djimdé AA, Diakité AA, Diawara M, Togo B, et al. Le paludisme congénital et néonatal en Afrique subsaharienne, un évènement rare? J Pédiatrie Puériculture. 2011;24(2):57-61.
21. Akindele JA, Sowunmi A, Abohweyere AEJ. Congenital malaria in a hyperendemic area: a preliminary study. Ann Trop Paediatr. 1993;13(3):273-6.
22. Ibhanesebhor SE. Clinical characteristics of neonatal malaria. J Trop Pediatr. 1995;41(6):330-3.
23. Orogade AA. Neonatal malaria in a mesoendemic malaria area of Northern Nigeria. Ann Afr Med. 2004;3(4):170-4.
24. Obiajunwa PO, Owa JA, Adeodu OO. Prevalence of congenital malaria in Ile-Ife. Nigeria. J Trop Pediatr. 2005;doi:10.1093/tropej/fmi003
25. Mukhtar MY, Lesi FEA, Iroha EU, Egri-Okwaji MTC, Mafe AG. Congenital malaria among inborn babies at a tertiary centre in Lagos, Nigeria. J Trop Pediatr. 2006; doi:10.1093/tropej/fmi044.
26. Okafor UH, Oguonu T, Onah HE. Risk factors associated with congenital malaria in Enugu, South Eastern Nigeria. J Obstet Gynaecol (Lahore). 2006;doi:10.1080/09638280600902893.
27. Falade C, Mokuolu O, Okafor H, Orogade A, Falade A, Adedoyin O, et al. Epidemiology of congenital malaria in Nigeria: a multi-centre study. Trop Med Int Health. 2007;doi:10.1111/j.1365-3156.2007.01931.x.
28. Sotimehin SA, Runsewe-Abiodun TI, Oladapo OT, Njokanma OF, Olanrewaju DM. Possible risk factors for congenital malaria at a tertiary care hospital in Sagamu, Ogun State, South-West Nigeria. J Trop Pediatr. 2008;doi:10.1093/tropej/fmn016.
29. Orogade AA, Falade CO, Okafor HU, Mokuolu OA, Mamman AI, Ogbonu TA, et al. Clinical and laboratory features of congenital malaria in Nigeria. J Pediatr Infect Dis. 2008;3(3):181-7.
30. Ekanem AD, Anah MU, Udo JJ. The prevalence of congenital malaria among neonates with suspected sepsis in Calabar, Nigeria. Trop Doct. 2008;38(2):73-6.
31. Lesi FEA, Mukhtar MY, Iroha EU, Egri-Okwaji MTC. Clinical presentation of congenital malaria at the Lagos University Teaching Hospital. Niger J Clin Pract. 2010;13(2):134-8.
32. Oduwole OA, Ejezie GC, Odey FA, Oringanje CM, Nwakanma D, Bello S, et al. Congenital malaria in Calabar, Nigeria: the molecular perspective. Am J Trop Med Hyg. 2011;doi:10.4269/ajtmh.2011.10-253.
33. Omalu ICJ, Mgbemena C, Mgbemena A, Ayanwale V, Olayemi IK, Lateef A, et al. Prevalence of congenital malaria in Minna, North Central Nigeria. J Trop Med. 2012;doi:10.1155/2012/274142.
34. George I. Prevalence of congenital malaria in Port Harcourt, Nigeria. Br J Med Res. 2014;doi:10.9734/bjmmr/2013/1436.
35. Silva H, Laulate B, Coral C. [Congenital malaria in a hospital in Iquitos, Peru]. Rev Peru Med Exp Salud Publica. 2015;32(2):259-64.
36. Omer SA, Noureldein AN, Eisa H, Abdelrahim M, Idress HE, Abdelrazig AM, et al. Impact of submicroscopic Plasmodium falciparum parasitaemia on maternal anaemia and low birth weight in Blue Nile State, Sudan. J Trop Med. 2019;doi:10.1155/2019/3162378.
37. Kitua AY, Smith T, Alonso PL, Masanja H, Urassa H, Menendez C, et al. Plasmodium falciparum malaria in the first year of life in an area of intense and perennial transmission. Trop Med Int Health. 1996;1(4):475-84.
38. Adachi M, Manji K, Ichimi R, Nishimori H, Shindo K, Matsubayashi N, et al. Detection of congenital malaria by polymerase-chain-reaction methodology in Dar es Salaam. Tanzania. Parasitol Res. 2000;doi:10.1007/pl00008540.
39. Mosha TCE, Ntarukimana D, John M. Prevalence of congenital malaria among neonates at Morogoro Regional Hospital, Morogoro, Tanzania. Tanzan J Health Res. 2010;doi:10.4314/thrb.v12i4.51792.
40. Obu H, Ibe B. Neonatal malaria in the gambia. Ann Med Health Sci Res. 2011;1(1):45-54.
41. Balaka B, Agbere AD, Bonkoungou P, Kessie K, Assimadi K, Agbo K. Paludisme congénital-maladie à Plasmodium falciparum chez le nouveau-né à risque infectieux. Arch Pédiatrie. 2000;7(3):243-8.
42. Nyirjesy P, Kavasya T, Axelrod P, Fischer PR. Malaria during pregnancy: neonatal morbidity and mortality and the efficacy of chloroquine chemoprophylaxis. Clin Infect Dis. 1993;16(1):127-32. doi:10.1093/clinids/16.1.127.
43. Bardají A, Martínez-Espinosa FE, Arévalo-Herrera M, Padilla N, Kochar S, Ome-Kaius M, et al. Burden and impact of Plasmodium vivax in pregnancy: a multi-centre prospective observational study. PLoS Negl Trop Dis. 2017;11(6):e0005606.
