# Supplementary material for: Malaria in children aged <6 months: a narrative review of current evidence, recommendations and practice gaps
Source: Trop Med Health. 2026 Apr 24;54:94. doi: 10.1186/s41182-026-00935-5 (PMC13195890; doi:10.1186/s41182-026-00935-5)
Supplement: Supplementary file 2 — Additional file 2. [file 41182_2026_935_MOESM2_ESM.docx]

**Additional File 2**

**Table.** List of selected case studies in children aged <6 months or weighing <5 kg

| 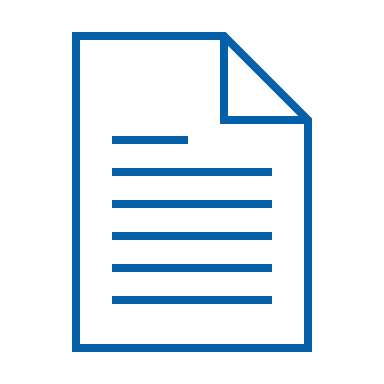 Study citation | 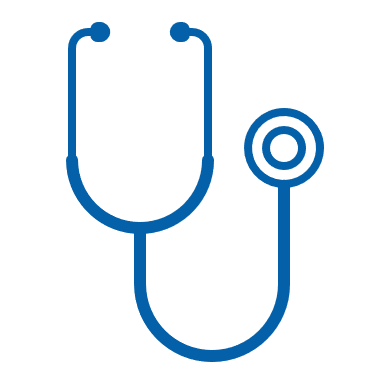 Clinical presentation | Diagnosis and treatment setting | Recommendation(s) |
| --- | --- | --- | --- |
| ***P. vivax* malaria** | | | |
| [Chandelia and Jain 2014](https://www.ncbi.nlm.nih.gov/pmc/articles/PMC4316305/) [1]  (Case report: India) | Co-infection of *P. vivax* malaria with CMV in a 30-day-old infant (BW of 2.7 kg) who presented with high-grade fever on and off for the past 7 days, with lethargy and decreased oral intake for 2 days | - In-patient monitoring - Screened for ToRCH, neonatal sepsis and haemolytic anaemia Repeat (48 hours) peripheral smear showed trophozoites of *P. vivax* while blood cell culture was again sterile - Context specific: Chloroquine was administered as a total dose of 25 mg/kg over 3 days^a^ - Follow-up of anaemia post-discharge | Malaria testing remains important if there is a possibility; it may occur as a comorbidity. |
| [Bugvi and Ahmed 2015](https://pubmed.ncbi.nlm.nih.gov/26577975/) [2]  (Case report: Pakistan) | An afebrile 6-week-old infant (4.6 kg) presented with a history of poor feeding of 20 days, pallor, jaundice and massive splenomegaly | - In-patient monitoring - A clinical diagnosis of jaundice with anaemia and hepatosplenomegaly was made initially - Laboratory-confirmed anaemia, thrombocytopenia and bilirubinaemia - Peripheral smear examination revealed parasitaemia - Off-label dihydroartemisinin:  15 mg, piperaquine phosphate:  120 mg once daily+syrup artem (artemether-lumefantrine)  1.5 mL/kg/day for 3 consecutive days | Peripheral smear examination should be performed in hospitalised patients who may be at risk of malaria. |
| [Bhatia et al. 2016](https://www.hindawi.com/journals/cripe/2016/1929046/) [3]  (Case Report: India) | A 6-day-old neonate with poor feeding, fever and loose stools for 2 days. The infant was pale, icteric and lethargic, with an axillary temperature of 100 °F, and liver and spleen were palpable 3 cm and 6 cm below the right and left subcostal margins, respectively | - In-patient monitoring - A provisional diagnosis of neonatal sepsis; the patient was started on IV ampicillin and gentamicin - The peripheral blood film revealed trophozoites and schizonts of *P. vivax*, with a parasite index of 2% - Contextual: Chloroquine base at a dose of 10 mg/kg stat followed by 5 mg/kg at 6, 24 and 48 hours | CM should be taken as a differential diagnosis for neonatal sepsis |
| [Tekle et al. 2018](https://www.ncbi.nlm.nih.gov/pmc/articles/PMC6169164/) [4]  (Case Report: Italy) | A preterm neonate (35 weeks and 5 days), weighing 2.5 kg, was born from an Eritrean mother with varicella zoster viral infection. During the NICU stay, the newborn presented with a febrile syndrome accompanied with vomiting, anaemia and thrombocytopenia | - In-patient monitoring - *P. vivax* trophozoites with 0.1% parasitaemia were detected in thick and thin blood smears - The diagnosis was confirmed by PCR assay and RDT - IV artesunate (2.4 mg/kg administered twice daily on the first day and then once daily for 3 days) and IV clindamycin (10 mg/kg as the loading dose and then 5 mg/kg every 8 hours for 7 days) were started | - Epidemiological risk assessment on malaria exposure in pregnant women traveling abroad from a malaria-endemic country - CM should be included in the differential diagnosis of infants displaying signs and symptoms of a febrile syndrome with haemolytic anaemia, thrombocytopenia or any other sepsis-like manifestation |
| ***P. falciparum* malaria** | | | |
| [Romani et al. 2018](https://malariajournal.biomedcentral.com/articles/10.1186/s12936-018-2614-9) [5]  (Case report: Italy) | A 2-month-old child was admitted due to anaemia and exposure to HIV. The baby was born prematurely by a caesarean section at 34 weeks’ gestation after a bicorial, biamniotic pregnancy by a migrant woman from Nigeria | - In-patient monitoring - Spleen and liver enlargement was observed in conjunction with anaemia - Maternal RDT sample was negative, while the infant RDT was positive; in both cases, microscopy of blood smears and PCR assay showed infection with *P. falciparum* - Off label: Oral administration of atovaquone/proguanil (125 mg/50 mg daily for 3 days) was immediately started | Malaria testing of infants born to mothers coming from areas of risk, as malaria can occur as a co-morbid condition |
| [Olupot-Olupot et al. 2018](https://doi.org/10.1186/s12936-018-2327-0) [6]  (Case series: East Uganda) | Four cases (two CM^b^ cases and two neonatal malaria^c^ cases). Three cases had fever and a history of fever, but one was hypothermic (34.8 °C) and had no history of fever. One case of CM had low birth weight, while the other was born to a HIV infection–positive mother. Both cases of CM presented with poor feeding; additionally, one of them had clinical jaundice. Neonatal malaria cases were born to primigravidae | - In-patient monitoring - Treatment with IV artesunate | Routine testing of neonates presenting with features of suspected sepsis in malaria-endemic settings |
| [Kajoba et al. 2021](https://www.hindawi.com/journals/criid/2021/9960006/) [7]  (Case report: Uganda) | A 2-day-old neonate presented with fever, irritability yellowing of the eyes and skin, and failure to breastfeed | - In-patient monitoring - The neonate had positive Giemsa-stained peripheral smear for *P. falciparum*, with a positive MRDT for *P. falciparum* malaria - Neonatal sepsis was suspected - IV artesunate was initiated - IV empiric antibiotics (cloxacillin and cefotaxime) were also administered for suspected neonatal sepsis | CM should be considered as a differential diagnosis for sepsis in neonates in malaria-endemic areas presenting with unexplained fever and ill health |
| [Nwaneli et al. 2022](https://malariajournal.biomedcentral.com/articles/10.1186/s12936-022-04056-2) [8]  (Case report: Nigeria) | A 5-day-old neonate born to an apparently healthy mother presented with a 3-day history of yellowness of the body and a 2-day history of poor breastfeeding and excessive sleep. Neonate developed characteristics consistent with meningitis | - In-patient monitoring - A presumptive diagnosis of early onset of neonatal sepsis and severe neonatal hyperbilirubinemia was made - A peripheral blood film showed malaria parasites, which was confirmed by Giemsa-stained thick and thin blood film microscopy for CCM - Parenteral artesunate followed by off-label oral artemisinin combination therapy | - CCM and infant meningitis have similar clinical presentations; CCM could be misdiagnosed - Cerebral malaria can present as a manifestation of CM and should be considered in patients who present with seizures and an unarousable coma - Sick neonates born to asymptomatic mothers in malaria-endemic areas should also be promptly investigated for malaria - Although additional data are needed in neonates, IV artesunate followed by oral artesunate–amodiaquine treatment appeared to be safe and effective |
| ^a^In reference to Guideline for diagnosis and treatment of Malaria in India 2009. Available at: <https://ncvbdc.mohfw.gov.in/Doc/Guidelines-Diagnosis-Treatment-Mal-2009.pdf>. Accessed 8 December, 2025.  ^b^Congenital malaria was defined as the direct infection of an infant with malaria parasites from their mother before or during birth.  ^c^Neonatal malaria refers to infections occurring within the first 8-28 days of life, resulting from an infective mosquito bite received after birth.  BW, body weight; CM, congenital malaria; CCM, congenital cerebral malaria; CMV, cytomegalovirus; HIV, human immunodeficiency virus; IV, intravenous; MRDT, malaria rapid diagnostic test; NICU, neonatal intensive care unit; PCR, polymerase chain reaction; RDT, rapid diagnostic test; ToRCH, toxoplasmosis, rubella cytomegalovirus, herpes simplex and HIV. | | | |

**References**

1. Chandelia S, Jain S. Co-infection of Plasmodium vivax malaria and cytomegalovirus in an immunocompetent neonate. J Clin Diagn Res. 2014;doi:10.7860/JCDR/2014/7615.5224.
2. Bugvi SM, Ahmed N. Congenital malaria: a rare entity. J Coll Physicians Surg Pak. 2015;25(11):841-2.
3. Bhatia R, Rajwaniya D, Agarwal P. Congenital malaria due to Plasmodium vivax infection in a neonate. Case Rep Pediatr. 2016;doi:10.1155/2016/1929046.
4. Gebremeskel Tekle S, Corpolongo A, D'Abramo A, Giancola ML, Iannetta M, Scorzolini L, et al. Case report: delayed diagnosis of congenital malaria by *Plasmodium vivax* in a newborn of an Eritrean woman with varicella infection. Am J Trop Med Hyg. 2018;99(3):620-2.
5. Romani L, Pane S, Severini C, Menegon M, Foglietta G, Bernardi S, et al. Challenging diagnosis of congenital malaria in non-endemic areas. Malar J. 2018;doi:10.1186/s12936-018-2614-9.
6. Olupot-Olupot P, Eregu EIE, Naizuli K, Ikiror J, Acom L, Burgoine K. Neonatal and congenital malaria: a case series in malaria endemic eastern Uganda. Malar J. 2018;doi:10.1186/s12936-018-2327-0.
7. Kajoba D, Ivan Egesa W, Jean Petit H, Omar Matan M, Laker G, Mugowa Waibi W, et al. Congenital malaria in a 2-day-old neonate: a case report and literature review. Case Rep Infect Dis. 2021;doi:10.1155/2021/9960006.
8. Nwaneli EI, Nri-Ezedi CA, Okeke KN, Edokwe ES, Echendu ST, Iloh KK. Congenital cerebral malaria: a masquerader in a neonate. Malar J. 2022;doi:10.1186/s12936-022-04056-2.
